# An Approach to Providing Timely Mental Health Services to Diverse Youth Populations

**DOI:** 10.1001/jamapsychiatry.2024.4880

**Published:** 2025-02-26

**Authors:** Srividya N. Iyer, Patricia Boksa, Ridha Joober, Jai Shah, Rebecca Fuhrer, Neil Andersson, Shalini Lal, Giuseppe D’Andrea, Nora Morrison, Valerie Noel, Daniel Rabouin, Tovah Cowan, Kathleen MacDonald, Mary Anne Levasseur, Feodor Poukhovski-Sheremetyev, Amal Abdel-Baki, Lacey Augustine, Kevin Friese, Isabelle Godin, Katherine Hay, Daphne Hutt-MacLeod, Vickie Plourde, Norma Rabbitskin, Paula Reaume-Zimmer, Cécile Rousseau, Heather Rudderham, Adam Abba-Aji, Diane Aubin, Liana Urichuk, Helen Vallianatos, Shirin Golchi, Ina Winkelmann, Jessica Chisholm-Nelson, Ashok Malla

**Affiliations:** 1 Department of Psychiatry, McGill University, Montreal, Quebec, Canada; 2 ACCESS Open Minds; 3 Prevention and Early Intervention Program for Psychosis, Douglas Mental Health University Institute, Montreal, Quebec, Canada; 4 Douglas Mental Health University Institute, Montreal, Quebec, Canada; 5 Department of Epidemiology, Biostatistics and Occupational Health, McGill University, Montréal, Québec, Canada; 6 Department of Family Medicine, Community Information and Epidemiological Technologies Institute and Participatory Research at McGill (PRAM), McGill University, Montréal, Québec, Canada; 7 McGill University Institute for Human Development and Well-being, Montréal, Québec, Canada; 8 School of Rehabilitation, Faculty of Medicine, Université de Montreal, Montreal, Quebec, Canada; 9 Centre de recherche du Centre hospitalier de l’Université de Montréal, Montréal, Québec, Canada; 10 ACCESS Open Minds Family and Carers Council, Douglas Mental Health University Institute, Montreal, Quebec, Canada; 11 ACCESS Open Minds Youth Council, Douglas Mental Health University Institute, Montreal, Quebec, Canada; 12 Department of Psychiatry, Université de Montréal, Montréal, Québec, Canada; 13 Centre hospitalier de l’Université de Montréal, Montréal, Québec, Canada; 14 Dans La Rue and Réseau d’intervention de proximité auprès des jeunes de la rue-Montréal/Homeless Youth Network, Montréal, Québec, Canada; 15 Elsipogtog Health and Wellness Centre and ACCESS Open Minds New Brunswick, Elsipogtog First Nation, New Brunswick, Canada; 16 Office of the Dean of Students, University of Alberta, Edmonton, Alberta, Canada; 17 ACCESS Open Minds University of Alberta, Edmonton, Alberta, Canada; 18 ACCESS Open Minds-Esprits ouverts New Brunswick, Acadian Peninsula, Moncton, New Brunswick, Canada; 19 Centre de Bénévolat de la Péninsule Acadienne, New Brunswick, Canada; 20 ACCESS Open Minds Edmonton, Edmonton, Alberta, Canada; 21 now with Kickstand Integrated Youth Services Initiative, Alberta, Canada; 22 ACCESS Open Minds Eskasoni First Nation, Eskasoni First Nation, Nova Scotia, Canada; 23 School of Psychology, University of Moncton, Moncton, New Brunswick, Canada; 24 Sturgeon Lake Health Centre, Sturgeon Lake First Nation, Saskatchewan, Canada; 25 ACCESS Open Minds Chatham-Kent, Chatham-Kent, Ontario, Canada; 26 Centre de recherche SHERPA, Institut Universitaire au regard des communautés culturelles, Centre intégré universitaire de santé et de services sociaux du Centre-Ouest-de-l’Île-de-Montréal, Montréal, Québec, Canada; 27 Department of Psychiatry, University of Alberta, Edmonton, Alberta, Canada; 28 PolicyWise for Children and Families, Alberta, Canada; 29 Department of Anthropology, University of Alberta, Edmonton, Alberta, Canada; 30 now with Nova Scotia Integrated Youth Services Initiative, Nova Scotia, Canada; 31 now with Nova Scotia Health, Sydney, Nova Scotia, Canada; 32 now with Bluewater Health, Sarnia, Ontario, Canada

## Abstract

**Importance:**

Accessing mental health care is challenging for youths, especially those facing intersectional disadvantages, but whether enhancing youth services increases reach and timeliness has rarely been investigated. ACCESS Open Minds (ACCESS-OM) transformed services at urban, rural, and Indigenous sites in Canada using 5 principles (early identification, rapid access, appropriate care, no age-based transitions from 11-25 years, and youth and family engagement).

**Objective:**

To evaluate whether the number of youths referred (hypothesis 1), offered evaluation appointments within 72 hours of referral (hypothesis 2), and receiving services within 30 days of the first appointment (hypothesis 3) increased over the course of ACCESS-OM’s implementation.

**Design, Setting, and Participants:**

This cohort study included youths (aged 11-25 years) at 11 sites referred between March 2016 and December 2020. Data were analyzed from April 2022 to April 2024.

**Exposure:**

Existing primary and/or community services implemented ACCESS-OM’s core components: broad-spectrum mental health services, outreach, youth-friendly walk-in spaces, open systems accepting referrals from multiple sources, and response-time benchmarks (72 hours to evaluation and 30 days to start treatment).

**Main Outcomes and Measures:**

Outcomes were the referral rate and the probability of being offered a first evaluation within 72 hours and receiving services within 30 days. Dates of referral and/or help-seeking, first offered appointment, first evaluation, and first services received were recorded. Multilevel negative binomial regression was used for hypothesis 1, and time-to-event analyses followed by multilevel accelerated failure time (AFT) models were used for hypotheses 2 and 3.

**Results:**

A total of 7889 youths were referred; 4519 (mean [SD] age, 19.3 [3.4] years; 2440 [54%] cisgender women; 1049 [23.21%] Indigenous; 991 [21.93%] Visible Minority [Arab, Black, Chinese, Filipino, Japanese, Korean, Latin American, South Asian, Southeast Asian, West Asian, other ethnicity, and multiple ethnicities]; and 1525 [49.10%] White) were evaluated before March 2020. Each 6-month progression after implementation was associated with a 10% increase in referral rates (IRR, 1.10; 95% CI, 1.07-1.13). The probability of being offered an initial appointment (χ^2^_2_ = 20.30; *P* < .001) and receiving services (χ^2^_2_ = 4.48; *P* = .01) after any given delay differed significantly over the 3 years. In adjusted AFT models, each 6-month progression was associated with a 3% decrease in time to offered evaluation (time ratio [TR], 0.97; 95% CI, 0.95-0.99) and first services (TR, 0.97; 95% CI, 0.94-1.00). Moderate to severe mental health problems were associated with longer delays to offered first appointments (TR, 1.14; 95% CI, 1.06-1.24) and services (TR, 1.11; 95% CI, 1.01-1.22).

**Conclusions and Relevance:**

As hypothesized, after ACCESS-OM implementation, more youths sought help, and the timeliness of initial response and services improved over time. These findings suggest that core principles, benchmarks, and implementation supports are valuable in organizing youth mental health care. Future efforts should make benefits equitable for those with severe problems.

## Key Points

**Question:**

Do reach and timeliness increase after existing primary and/or community youth mental health services are enhanced?

**Findings:**

In this cohort study of 4519 youths, the number of referrals and the timeliness of the initial evaluation and interventions increased over time after youth mental health services were enhanced. Youths with moderate to severe mental health problems experienced longer delays.

**Meaning:**

These findings suggest that low-barrier, primary and/or community, holistic services for youths with varying presentations are promising, and that further work is needed to determine why some youths, like those with severe problems, are not benefiting equitably and to train services to respond faster to them and integrate better with specialist services.

## Teaser

This cohort study evaluates whether an intervention in youth services in Canada increased the numbers of youth referred, appointments scheduled within 72 hours of referral, and youths receiving services within 30 days of the first appointment.

## Introduction

Mental health problems, 68% of which emerge before the age of 25 years, are leading causes of disability and death in youths.^1,2,3^ They can be worsened by delayed or poor-quality treatment^3^ and have serious long-term consequences.^3^ Yet, youths often face complex pathways to mental health care, long waits, siloed care, developmentally and culturally insensitive care, and poor outcomes.^3,4,5,6,7^ These problems are especially common for marginalized groups^8,9,10^ like racial and ethnic minority and homeless youths. In countries with histories of settler colonialism, Indigenous youths face additional challenges due to intergenerational trauma, poverty, geographical isolation, and so forth.^10^

There is growing consensus around investing in youth mental health.^3,6^ In Canada, large-scale reform was spearheaded by ACCESS Open Minds (ACCESS-OM),^4,7,11,12^ a project involving youths; families; Indigenous, community and health care partners; researchers; and decision-makers. ACCESS-OM developed, implemented, and evaluated a youth mental health service transformation across urban, rural, remote, and Indigenous sites. Two sites served postsecondary students and youths experiencing homelessness. Existing services in primary and/or community settings were enhanced along 5 principles identified through a theory of change^4,13^: early identification; rapid access; appropriate, culturally relevant care; no age-based transitions; and involving youths and families or carers in service and care design.^14,15^ These principles were flexible by design, so their operationalization could be tailored to local cultural and contextual factors (see special issue with descriptions of diverse sites in separate publications).^12^

Drawing on Australian and Irish initiatives^3^ and our team’s experience with early psychosis intervention,^16^ ACCESS-OM addressed known systemic shortcomings, including long wait times and lists^17^; multistep pathways (eg, general practitioners gatekeeping access to mental health care)^5^; diagnoses-based exclusions^18^; disjointed transitions between child-adolescent and adult services^3,6^; and minimal stakeholder involvement.^19^

Core components implemented at all sites^7^ included offering services to youths aged 11 to 25 years experiencing all types and severities of mental health problems; targeted outreach to increase referrals; community mapping to identify and integrate youth-focused services via colocation or partnership; well-publicized, youth-friendly walk-in spaces in accessible locations created and/or renovated with ACCESS-OM funds; accepting referrals from multiple sources (self, family, peers, schools, physicians, Elders, and so forth); and response time benchmarks.

At all sites, benchmarks^7^ (eFigure 1 in Supplement 1) included offering appointments within 72 hours of referrals with nonphysician professional(s), who were trained to conduct initial, engaging evaluations with youths and link them with appropriate services. This benchmark, long used by Canadian early psychosis services,^20^ was deemed acceptable by stakeholders. Services or interventions were required to be started within 30 days of the first appointment, as recommended by the Canadian Psychiatric Association for nonurgent cases.^21^ This was a vast improvement over the Canadian status quo of wait times from 45 days to 1.5 years.^17,22^

All youths were offered an evaluation and mental health and substance use interventions (on site and/or navigated to external specialist services), based on their needs and preferences, clinicians’ evaluations, and service availability. Valuing holism, sites also offered peer support, Indigenous programming, supported employment, housing, and so forth. Coordinated by a central office, capacity was added at all sites through a yearly plan outlining how existing resources, new staff, and partnerships would be deployed to enact ACCESS-OM’s 5 objectives; training and capacity-building; and data-informed quality improvement discussions.

Few evaluations of youth services reform have tested whether they increased reach and reduced wait times, despite these being common problems and policy priorities.^3,5,17,22^ To address this gap, the primary hypotheses of our multistakeholder-codesigned research^7^ were that ACCESS-OM implementation would increase the number of youths who were referred, offered appointments within 72 hours of referral, and received services within 30 days of the first appointment. Delays were examined separately for youths with and without moderate to severe mental health problems, given concerns about youths with more serious problems having poorer outcomes in primary and/or community-based services.^23^

## Methods

### Design and Setting

The Douglas Research Centre’s research ethics board and local institutional, community and Indigenous bodies approved the study. ACCESS-OM follows Ownership, Control, Access, and Possession principles and Tri-Council guidelines for research involving Indigenous peoples.^24,25^ All sites and the ACCESS-OM Indigenous council coconceived and approved this research. Earlier publications^26^ detail ACCESS-OM’s governance and monitoring. The study followed Strengthening the Reporting of Observational Studies in Epidemiology (STROBE) reporting guidelines.

Twelve sites in small (1 site), medium (1 site), urban (6 sites) population centers, and Indigenous communities (4 sites) across 6 of Canada’s 10 provinces participated in a longitudinal cohort study. Sites represent variations in geography, culture, structure, resources, and population density (site descriptions^12^ and protocol^7^ previously published). Along with this cohort study, ACCESS-OM’s protocol included a stepped-wedge randomized clinical trial (RCT) involving only 6 sites. A cohort study and a cluster randomized trial had been chosen as tightly controlled traditional RCTs are difficult to justify given consensus on the urgency of reform. This report focuses on ACCESS-OM’s cohort study. The stepped-wedge trial was abandoned because its timelines were rendered unfeasible by policy changes (including fundamental services reorganization in 1 province) and systemic challenges. This decision was supported by ACCESS-OM’s steering and research committees, who were sensitive about pressuring communities, particularly 2 Indigenous ones in the trial. Still, our study adds a measure of rigor to youth mental health reform evaluations by testing a priori hypotheses in diverse primary and community settings and analyzing changes over time, both actions that have rarely been taken before.

### Procedure

All youths referred to or seeking help at ACCESS-OM sites were eligible for study inclusion. Each site recorded its monthly referral numbers. Absent referral data, the number of youths evaluated or consenting to the study were used as conservative proxies. For each participant, staff recorded the dates of referral, first offered appointment, first evaluation, and first services received, along with type(s) of services received, including on-site services (eg, counseling or therapy) and off-site specialist services (eg, for first-episode psychosis).

During the initial evaluation, youths self-reported sociodemographic data, including ethnic or cultural origins for which Statistics Canada's response options for the 2016 census^27^ were used and youth were grouped as Indigenous, Visible Minority or White as in its reports.^28,29^ Staff assessed the severity of mental health problems using the transdiagnostic version of the Clinical Global Impressions (CGI),^30^ a single-item, 7-point scale. Aligned with the scale’s anchors, a score of 1 is “no mental health problems”; 2, “borderline mental health problems”; 3, “mild mental health problems”; and scores from 4 to 7 indicate “moderate” to “extremely severe” problems. Staff received similar training and calibrated ratings against one another and expert clinician-scientists during booster sessions by arriving at consensus ratings of case vignettes. A secure electronic system facilitated multisite data collection and centralized data management.

### Statistical Analysis

Analyses were performed using R version 3.6.3 (R Project for Statistical Computing)^31^ and Stata version 18 (StataCorp).^32^ Only pre–COVID-19 data were included to avoid capturing pandemic-related anomalies. An Indigenous site (33 participants) that did not collect data on primary outcomes was excluded, leaving 11 sites in the cohort study.

Missing data were handled via multivariate imputation by chained equations in R.^33^ All variables and relevant auxiliary variables were imputed. All analyses were conducted post imputation on 60 datasets and results pooled with Rubin Rules. The nature, proportion, and handling of missing data are presented in eTable 1 in Supplement 1.

To test hypothesis 1, regarding increases in referrals, negative binomial regressions were fitted with referrals per month as dependent variable and time (6-month intervals) as independent variable. A multilevel approach with random intercepts by site (11 sites) was adopted. Each catchment’s at-risk population, youths aged 11 to 25 years, was estimated from census and demographic data.^7,34^ The population was multiplied by study duration (29-48 months) to estimate person-months at-risk, which were then added to the model as an offset. A negative binomial model was chosen over Poisson model, based on dispersion parameters (0.98 and 3.64, respectively) and better fit (χ^2^_1_ = 603.36; *P* < .001). The unadjusted incidence rate ratio (IRR) and the estimated mean for every 6 months of ACCESS-OM implementation were calculated.

To test hypothesis 2, regarding increases in numbers of youths offered initial appointments within 72 hours, Kaplan-Meier curves comparing time from referral to first offered appointment during the first 3 implementation years were estimated. The log-rank test was used to examine whether time to first offered appointment differed significantly between program years. Accelerated failure time (AFT) models were fitted to estimate changes in times to first appointment in relation to calendar time (6-month intervals from start of implementation). Covariates—age; ethnic and cultural origins; gender; sexual orientation; ability to meet basic needs; not in education, employment, or training (NEET) status; and moderate to severe mental health problems—were chosen a priori, based on literature and theory. Analyses were restricted to 42 months because few sites had data beyond then. Adopting a parametric approach, survival time was assumed to follow a known distribution. After evaluating exponential, Weibull, loglogistic, and log-normal distributions using Akaike Information Criterion (AIC), AFT models were fitted assuming a log-normal distribution to estimate time ratios (TRs) with 95% CIs.

To test hypothesis 3, regarding increases in numbers of youths being offered services within 30 days of first appointment, we again conducted Kaplan-Meier analyses followed by AFT models. The best-fitting distribution (log-normal) was determined based on AIC.

All AFT models were multilevel mixed-effects models with random intercepts by site. Random effects were assumed to follow a normal distribution. Statistical significance was determined through examining 95% confidence intervals. Data were analyzed from April 2022 to April 2024.

## Results

### Participants

The Figure describes sample sizes. From March 2016 to December 2020, 7889 youths were referred to the 11 sites. Of them, 4519 (mean [SD] age, 19.3 [3.4] years; 2440 [54%] cisgender women; 1049 [23.21%] Indigenous; 991 [21.93%] Visible Minority [Arab, Black, Chinese, Filipino, Japanese, Korean, Latin American, South Asian, Southeast Asian, West Asian, other ethnicity, and multiple ethnicities]; and 1525 [49.10%] White) received an initial evaluation before March 1, 2020 (sociodemographic and clinical characteristics in Table 1). The sample included youths who were gender-diverse (n = 292 [6.47%]); sexual minority (n = 1764 [39.01%]); NEET (n = 1577 [34.87%]); and facing difficulties meeting basic needs (n = 1771 [39.18%]). Nearly two-thirds (n = 2872 [63.55%]) presented with moderate to severe mental health problems; 1036 (22.9%) with mild and 611 (13.52%) with no or borderline mental health problems. The study obtained data from 4519 of 6662 (67.8%) of the referred youths who had an initial evaluation.

**Figure.**
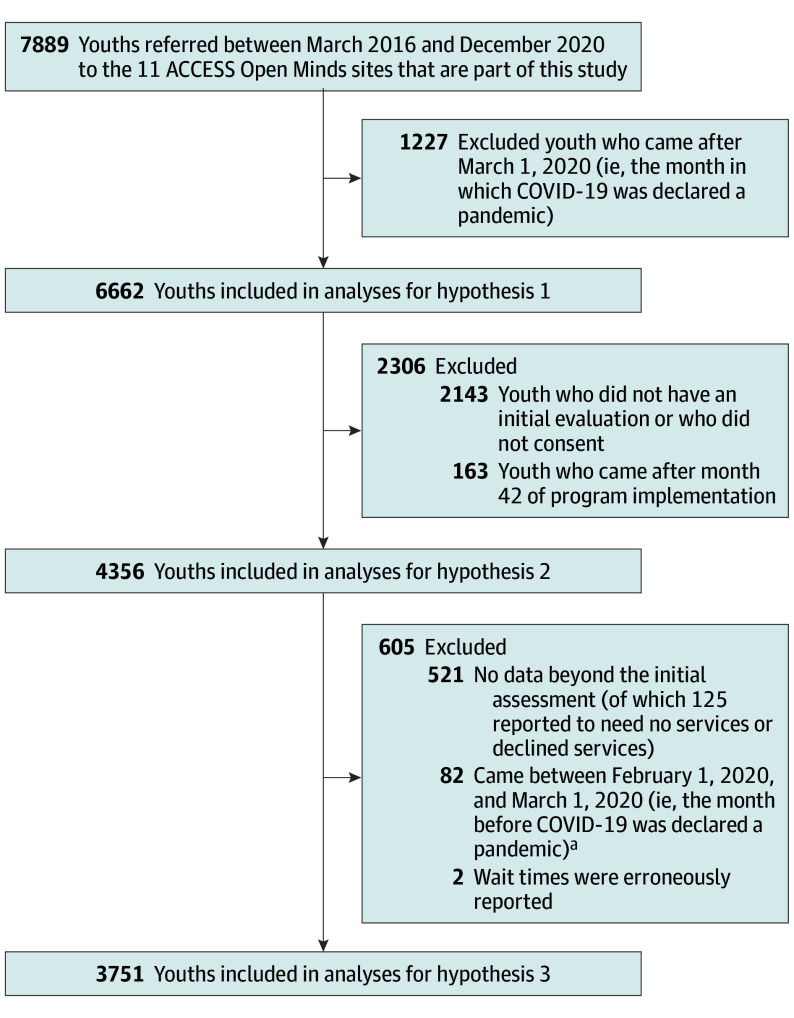
Flowchart Describing Sample Sizes for Each Hypothesis ^a^This was done to ensure that the study’s 30-day benchmark period occurred before the pandemic.

**Table 1.** Participant Characteristics Preimputation and Postimputation (N = 4519)^a^

Variable	Individuals, No. (%)	
	Preimputation	Postimputation
Age, y^b^		
Total No.	4324	4519
Mean (SD)	19.31 (3.40)	19.36 (3.38)
11-15	564/4324 (13.04)	574/4519 (12.70)
16-18	1230/4324 (28.45)	1269/4519 (28.07)
19-21	1305/4324 (30.18)	1376/4519 (30.44)
22-25	1225/4324 (28.33)	1301/4519 (28.79)
Gender		
Total No.	4255	4519
Cisgender woman	2307/4255 (54.22)	2440/4519 (53.99)
Cisgender man	1680/4255 (39.48)	1787/4519 (39.54)
Gender diverse^d^	268/4255 (6.30)	292/4519 (6.47)
Ethnic or cultural origins		
Total No.	3106	4519
Indigenous^c^	855/3106 (27.53)	1049/4519 (23.21)
Visible Minority^e^	726/3106 (23.37)	991/4519 (21.93)
White	1525/3106 (49.10)	2479/4519 (54.86)
Education, employment, and training status		
Total No.	2706	4519
Engaged in education, employment, or training	1824/2706 (67.41)	2944/4519 (65.13)
Not in education, employment, or training	882/2706 (32.59)	1577/4519 (34.87)
Ability to meet basic needs^f^		
Total No.	2264	4519
No difficulty meeting basic needs	1334/2264 (58.92)	2749/4519 (60.82)
Difficulty meeting basic needs	930/2264 (41.08)	1771/4519 (39.18)
Sexual orientation		
Total No.	2419	4519
Heterosexual	1467/2419 (60.64)	2757/4519 (60.99)
Sexual minority^g^	952/2419 (39.36)	1764/4519 (39.01)
Severity of mental health problems^h^		
Total No.	3337	4519
No to mild mental health problems	1168/3337 (35.00)	1647/4519 (36.45)
Moderate to severe mental health problems	2169/3337 (65.00)	2872/4519 (63.55)

^a^ The sample of 4519 reflects all young people from included sites who received an initial evaluation between July 2016 and March 2020.

^b^ Edmonton and University of Alberta do not include participants aged 11 to 15 years.

^c^ Preimputation, 7.5%, and postimputation, 11.9% of Indigenous youth accessed services from non-Indigenous sites.

^d^ Gender diverse includes transgender woman, transgender man, gender fluid, and I don’t identify with these options and prefer not to answer.

^e^ Visible Minority preimputation included Arab (n = 69 [9.5%]), Black (n = 172 [23.7%]), Chinese (n = 58 [8.0%]), Filipino (n = 43 [5.9%]), Japanese (n = 4 [0.6%]), Korean (n = 3 [0.4%]), Latin American (n = 78 [10.7%]), South Asian (n = 99 [13.6%]), Southeast Asian (n = 20 [2.8%]), West Asian (n = 17 [2.3%]), other ethnicity (n = 111 [15.3%]), and multiple ethnicities (n = 52 [7.2%]).

^f^ Basic needs includes access to food, shelter, and clothing.

^g^ Sexual minority included asexual, bisexual, gay, lesbian, not sure, questioning, queer, 2-Spirit, I prefer not to answer this question, and other.

^h^ Clinical Global Impression of Severity scores 1 to 3 are indicative of no to mild mental health problems, and 4 to 7 are indicative of moderate to severe mental health problems. A total of 148 (3%) scored 1 (no mental health problems), 463 (10%) scored 2 (borderline mental health problems), and 1036 (24%) scored 3 (mild mental health problems).

### Hypothesis 1

In the multilevel negative binomial regression (6662 youths referred before March 2020), accounting for person-months at risk at different sites, each 6-month progression after implementation was associated with an approximately 10% increase in the rate of referrals and self-referrals (IRR, 1.10; 95% CI, 1.07-1.13; *P* < .001). The marginal estimated mean of referrals increased from 8.29 (95% CI, 4.56-15.06) in the first 6 months to 15.91 (95% CI, 8.71-29.06) in the last 6 months (eFigure 2 in Supplement 1). There was substantial intersite heterogeneity (σ^2^ = 9.37).

### Hypothesis 2

Table 2 presents mean and median delays from referral to first offered appointment by sociodemographic and clinical characteristics (4356 youths evaluated before March 2020 and came in the first 42 months). eFigure 3 in Supplement 1 shows the Kaplan-Meier estimated probabilities of being offered an initial appointment after a delay of any given number of days following referral or help-seeking over the first 3 implementation years. During year 1, the cumulative probability of being offered an appointment by 72 hours was 0.48 (SE, 0.03). It increased to 0.62 (SE, 0.03) in year 2 and 0.64 (SE, 0.03) in year 3. A considerable reduction in wait times was achieved after year 1 and then sustained (χ^2^_2_ = 20.30; *P* < .001).

**Table 2.** Delay From Referral to Initial Offered Appointment by Sociodemographic and Clinical Characteristics of the Sample

Variable	Individuals, No. (%)^a^	Mean (SD), d	Median (IQR), d
Total sample^b^	4356 (100)	10.38 (28.1)	0.00 (0.00-13.62)
Time, mo			
1-6	380 (8.72)	18.22 (41.31)	3.06 (0.00-13.62)
7-12	481 (11.04)	14.77 (32.62)	3.00 (0.00-11.42)
13-18	669 (15.36)	9.70 (23.14)	0.00 (0.00-7.23)
19-24	666 (15.29)	8.85 (22.59)	0.00 (0.00-9.19)
25-30	793 (18.20)	9.01 (29.48)	0.00 (0.00-8.00)
31-36	752 (17.26)	9.73 (30.98)	0.00 (0.00-5.87)
37-42	615 (14.12)	7.91 (20.75)	0.00 (0.00-6.00)
Age, y			
11-15	541 (12.41)	19.20 (31.49)	8.10 (1.33-21.81)
16-18	1207 (27.71)	10.05 (24.00)	1.33 (0.00-8.84)
19-21	1335 (30.65)	8.41 (27.56)	0.00 (0.00-4.62)
22-25	1273 (29.22)	9.42 (31.60)	0.00 (0.00-5.05)
Gender			
Cisgender woman	2341 (53.75)	11.04 (28.09)	1.00 (0.00-8.65)
Cisgender man	1729 (39.68)	10.22 (29.21)	0.00 (0.00-7.12)
Gender diverse^c^	286 (6.57)	7.80 (29.11)	0.00 (0.00-3.73)
Ethnicity			
Indigenous	998 (22.92)	12.81 (30.95)	1.87 (0.00-11.74)
Visible Minority^d^	981 (22.53)	13.58 (32.64)	0.20 (0.00-11.42)
White	2376 (54.55)	8.25 (25.42)	0.00 (0.00-6.12)
Education, employment, or training status			
Engaged in education, employment, or training	2822 (64.79)	11.84 (29.51)	1.08 (0.00-10.18)
Not in education, employment, or training	1534 (35.21)	8.04 (26.65)	0.00 (0.00-4.25)
Ability to meet basic needs^e^			
No difficulty meeting basic needs	2628 (60.32)	11.50 (28.97)	0.97 (0.00-9.28)
Difficulty meeting basic needs	1729 (39.68)	8.97 (27.91)	0.00 (0.00-6.65)
Sexual orientation			
Heterosexual or straight	2651 (60.86)	11.06 (28.73)	0.35 (0.00-8.53)
Sexual minority^f^	1705 (39.14)	9.63 (28.35)	0.00 (0.00-6.93)
Severity of mental health problems^g^			
No to mild mental health problems	1570 (36.05)	9.11 (24.16)	0.97 (0.00-7.97)
Moderate to severe mental health problems	2786 (63.95)	11.28 (30.83)	0.00 (0.00-7.97)

^a^ Numbers represent the average across 60 imputed datasets rounded to the nearest whole number, but proportions were calculated before rounding.

^b^ A total of 60.4% (95% CI, 58.9%-61.9%) of the sample met the 72-hour benchmark with proportions ranging from 12.3% to 96.9% across sites.

^c^ Gender diverse included transgender woman, transgender man, gender fluid, and I don’t identify with these options and prefer not to answer.

^d^ Visible Minority preimputation included Arab (n = 69 [9.5%]), Black (n = 172 [23.7%]), Chinese (n = 58 [8.0%]), Filipino (n = 43 [5.9%]), Japanese (n = 4 [0.6%]), Korean (n = 3 [0.4%]), Latin American (n = 78 [10.7%]), South Asian (n = 99 [13.6%]), Southeast Asian (n = 20 [2.8%]), West Asian (n = 17 [2.3%]), other ethnicity (n = 111 [15.3%]), and multiple ethnicities (n = 52 [7.2%]).

^e^ Basic needs includes access to food, shelter, and clothing.

^f^ Sexual minority included asexual, bisexual, gay, lesbian, not sure, questioning, queer, 2-Spirit, I prefer not to answer this question, and other.

^g^ Clinical Global Impression of Severity scores 1 to 3 are indicative of no to mild mental health problems, and 4 to 7 are indicative of moderate to severe mental health problems. A total of 148 (3%) scored 1 (no mental health problems), 463 (10%) scored 2 (borderline mental health problems), and 1036 (24%) scored 3 (mild mental health problems).

In the adjusted AFT model (Table 3), every 6-month progression was associated with an approximately 3% decrease in delay to the first offered appointment (TR, 0.97; 95% CI, 0.95-0.99). Moderate to severe mental health problems were associated with 14% longer wait times (TR, 1.14; 95% CI, 1.06-1.24). No other association was found, except for a small one with age (TR, 0.99; 95% CI, 0.97-1.00), with younger individuals having longer wait times.

**Table 3.** Results of the Accelerated Time Failure Models

Variable	Adjusted TR (95% CI)^a^	
	Time from referral to first offered evaluation appointment	Time from first appointment to first service received
Referral period per 6-mo increment	0.97 (0.95-0.99)^b^	0.97 (0.94-1.00)^b^
Age, y, continuous	0.99 (0.97-1.00)	1.01 (0.99-1.03)
Gender		
Cisgender woman	1 [Reference]	1 [Reference]
Cisgender man	1.00 (0.93-1.08)	1.00 (0.91-1.10)
Gender diverse^c^	1.04 (0.89-1.21)	1.08 (0.91-1.29)
Ethnic or cultural origins		
Indigenous	1.04 (0.91-1.20)	0.92 (0.79-1.07)
Visible Minority^d^	1.03 (0.92-1.14)	0.97 (0.86-1.10)
White	1 [Reference]	1 [Reference]
Education, employment, or training status		
Engaged in education, employment, or training	1 [Reference]	1 [Reference]
Not in education, employment, or training	0.94 (0.84-1.05)	0.99 (0.89-1.10)
Ability to meet basic needs^e^		
No difficulty meeting basic needs	1 [Reference]	1 [Reference]
Difficulty meeting basic needs	0.96 (0.84-1.09)	0.97 (0.87-1.09)
Sexual orientation		
Heterosexual	1 [Reference]	1 [Reference]
Sexual minority^f^	0.94 (0.84-1.05)	1.06 (0.95-1.17)
Severity of mental health problems^g^		
No to mild mental health problems	1 [Reference]	1 [Reference]
Moderate to severe mental health problems	1.14 (1.06-1.24)^b^	1.11 (1.01-1.22)^b^

Abbreviation: TR, time ratio.

^a^ All models were mixed-effects multilevel models with random intercepts by site (n = 11).

^b^ Indicates statistically significant association.

^c^ Gender diverse included transgender woman, transgender man, gender fluid, and I don’t identify with these options and prefer not to answer.

^d^ Visible Minority preimputation included Arab (n = 69 [9.5%]), Black (n = 172 [23.7%]), Chinese (n = 58 [8.0%]), Filipino (n = 43 [5.9%]), Japanese (n = 4 [0.6%]), Korean (n = 3 [0.4%]), Latin American (n = 78 [10.7%]), South Asian (n = 99 [13.6%]), Southeast Asian (n = 20 [2.8%]), West Asian (n = 17 [2.3%]), other ethnicity (n = 111 [15.3%]), and multiple ethnicities (n = 52 [7.2%]).

^e^ Basic needs includes access to food, shelter, and clothing.

^f^ Sexual minority included asexual, bisexual, gay, lesbian, not sure, questioning, queer, 2-Spirit, I prefer not to answer this question, and other.

^g^ Clinical Global Impression of Severity scores 1 to 3 are indicative of no to mild mental health problems, and 4 to 7 are indicative of moderate to severe mental health problems. A total of 148 (3%) scored 1 (no mental health problems), 463 (10%) scored 2 (borderline mental health problems), and 1036 (24%) scored 3 (mild mental health problems).

### Hypothesis 3

Table 4 presents mean and median times from first appointment to first offered services (3751 youths with data beyond the evaluation and who came before February 2020). eFigure 4 in Supplement 1 shows the Kaplan-Meier estimated probabilities of receiving services after any given number of days following the first appointment over the first 3 years. Between the first and subsequent years, more people were likely to receive services with shorter delays (χ^2^_2_ = 4.48; *P* = .01). During year 1, the cumulative probability of receiving services within 30 days was 0.85 (SE, 0.09). It increased slightly during year 2 (0.86; SE, 0.07) and year 3 (0.89; SE, 0.08). The probability of receiving services on the day of the first appointment increased from 68% (SE, 0.05) in year 1 to 79% (SE, 0.05) in year 3.

**Table 4.** Delay From First Appointment (for an Evaluation) to First Received Services by Sociodemographic and Clinical Characteristics of the Sample

Variable	Individuals, No. (%)^a^	Mean (SD), d	Median (IQR)
Total sample^b^	3751 (100)	15.17 (50.80)	0.00 (0.00-0.00)
Time, mo			
1-6	348 (9.28)	20.30 (66.14)	0.00 (0.00-10.53)
7-12	381 (10.16)	17.40 (48.64)	0.00 (0.00-5.85)
13-18	510 (13.60)	23.33 (67.48)	0.00 (0.00-6.26)
19-24	612 (16.32)	13.91 (51.00)	0.00 (0.00-0.00)
25-30	717 (19.11)	16.70 (51.84)	0.00 (0.00-0.00)
31-36	689 (18.37)	9.94 (35.62)	0.00 (0.00-0.00)
37-42	494 (13.17)	5.95 (27.13)	0.00 (0.00-0.00)
Age, y			
11-15	468 (12.47)	26.81 (67.87)	0.00 (0.00-13.67)
16-18	1016 (27.08)	15.65 (49.16)	0.00 (0.00-0.89)
19-21	1149 (30.62)	12.36 (46.25)	0.00 (0.00-0.00)
22-25	1119 (29.83)	12.77 (47.35)	0.00 (0.00-0.00)
Gender			
Cisgender woman	2029 (54.10)	15.79 (49.39)	0.00 (0.00-0.54)
Cisgender man	1470 (39.19)	13.95 (48.38)	0.00 (0.00-0.00)
Gender diverse^c^	251 (6.70)	17.41 (71.30)	0.00 (0.00-0.00)
Ethnicity			
Indigenous	869 (23.17)	14.85 (49.92)	0.00 (0.00-0.00)
Visible Minority^d^	843 (22.47)	19.92 (55.29)	0.00 (0.00-6.66)
White	2039 (54.36)	11.33 (47.57)	0.00 (0.00-0.00)
Education, employment, or training status			
Engaged in education, employment, or training	2414 (64.35)	17.43 (53.02)	0.00 (0.00-1.31)
Not in education, employment, or training	1337 (35.65)	11.11 (46.19)	0.00 (0.00-0.00)
Ability to meet basic needs^e^			
No difficulty meeting basic needs	2249 (59.95)	16.60 (52.43)	0.00 (0.00-0.50)
Difficulty meeting basic needs	1502 (40.05)	13.04 (48.07)	0.00 (0.00-0.00)
Sexual orientation			
Heterosexual or straight	2281 (60.80)	14.76 (49.97)	0.00 (0.00-0.00)
Sexual minority^f^	1471 (39.20)	15.81 (51.98)	0.00 (0.00-0.12)
Severity of mental health problems^g^			
No to mild mental health problems	1344 (35.83)	12.55 (45.61)	0.00 (0.00-0.00)
Moderate to severe mental health problems	2407 (64.17)	16.64 (53.39)	0.00 (0.00-0.20)

^a^ Numbers represent the average across 60 imputed datasets rounded to the nearest whole number but proportions were calculated before rounding.

^b^ A total of 3751 youth with data who came before February 2020. This cutoff was chosen to allow for the 30-day benchmark period to be before the COVID-19-pandemic. A total of 87.83% (95% CI, 86.62%-89.04%) of the sample met the 30-day benchmark with proportions ranging from 23.54% to 97.22% across sites over the implementation years.

^c^ Gender diverse included transgender woman, transgender man, gender fluid, and I don’t identify with these options and prefer not to answer.

^d^ Visible Minority preimputation included Arab (n = 69 [9.5%]), Black (n = 172 [23.7%]), Chinese (n = 58 [8.0%]), Filipino (n = 43 [5.9%]), Japanese (n = 4 [0.6%]), Korean (n = 3 [0.4%]), Latin American (n = 78 [10.7%]), South Asian (n = 99 [13.6%]), Southeast Asian (n = 20 [2.8%]), West Asian (n = 17 [2.3%]), other ethnicity (n = 111 [15.3%]), and multiple ethnicities (n = 52 [7.2%]).

^e^ Basic needs includes access to food, shelter, and clothing.

^f^ Sexual minority included asexual, bisexual, gay, lesbian, not sure, questioning, queer, 2-Spirit, I prefer not to answer this question, and other.

^g^ Clinical Global Impression of Severity scores 1 to 3 are indicative of no to mild mental health problems, and 4 to 7 are indicative of moderate to severe mental health problems. A total of 148 (3%) scored 1 (no mental health problems), 463 (10%) scored 2 (borderline mental health problems), and 1036 (24%) scored 3 (mild mental health problems).

In the adjusted AFT model (Table 3), every 6-month progression was associated with an approximately 3% decrease in wait times from first appointment to first service (TR, 0.97; 95% CI, 0.94-1.00). Among covariates, only having a moderate to severe mental health problem was associated with 11% longer wait times to services (TR, 1.11; 95% CI, 1.01-1.22).

The top 7 first-received services (eTables 2 and 3 in Supplement 1) were individual therapy (mean [SD] delay to service, 13.4 [44.5] days); single-session therapy and care coordination during first appointment (mean [SD] delay to service, 0.0 [0.0] days); case management or care coordination (mean [SD] delay to service, 8.34 [43.4] days); psychosocial services (mean [SD] delay to service, 25.2 [54.4] days); peer support (mean [SD] delay to service, 8.5 [36.3] days); physical health care (mean [SD] delay to service, 7.0 [22.7] days); and psychiatric services (mean [SD] delay to service, 32.9 [61.7] days).

### Post Hoc Analyses

Because moderate to severe mental health problems were associated with delays to first offered appointment and service provision, adjunctive analyses introduced an interaction term between time since implementation and CGI score in AFT models. Median wait times to first offered appointment and services decreased over time after implementing ACCESS-OM independently of CGI scores (eFigure 5 in Supplement 1). Wait times were low from the beginning for those not at all to mildly ill and improved constantly for those with more severe problems. The interaction term did not improve the models according to the pooled likelihood ratio tests.

AFT models were repeated to compare differences in delays in being offered an initial appointment and receiving services among those with moderate to severe vis-à-vis mild and no or borderline mental health problems (eTable 4 in Supplement 1). Compared with those with moderate to severe problems, those with no or borderline mental health problems had 15% shorter wait times to the first offered appointment and 14% shorter wait times to services. Those with mild mental health problems had 12% shorter wait times to the first offered appointment. They also had shorter wait times to services, but this was not significant. eTable 5 in Supplement 1 presents mean and median delays by severity of mental health problems.

## Discussion

As hypothesized, after ACCESS-OM implementation, increasing numbers of youths accessed services, were offered an initial appointment, and received services with shorter delays. As envisioned, youths in need of mental health services accessed ACCESS-OM, with two-thirds presenting with moderate to severe mental health problems and only 13.5% with no or borderline mental health problems.

### Increased Access

The increase in youths seeking help did not plateau for the observation interval (29-48 months). This increase may be attributable to outreach and early identification activities and codesigned services but may also reflect secular trends in youth mental health problems and service utilization.^3^ Previous studies have reported postreform increases in referrals, but without a priori hypotheses, analyses of time trends, or accounting for varying numbers of youth at risk at sites. In Australia, increased youth mental health care usage from 2006 to 2008 was attributed to the Headspace initiative launched in 2006.^35^ In Birmingham, there were more referrals from months 6 to 12 after reform than in months 1 to 6.^36^ A Norfolk study reported a 68% increase in the first year over the preimplementation year.^37^ These studies and ours did not have a comparator, precluding conclusions about whether new models or their elements directly led to increased uptake.

Lesbian, gay, bisexual, transgender, queer, intersex, and 2-Spirit; NEET; racial and ethnic minority; and impoverished youths are usually underserved and have trouble accessing mental health care.^3,5,7^ Yet, they constituted a substantial share of ACCESS-OM clientele, equaling or exceeding their proportions in the general population.^38,39,40^ Marginalized youths may avail care when it is well publicized, accessible, youth friendly, adapted, holistic, and in primary and/or community settings. Future research should evaluate this assumption. The wide site-wise variance in increases in referrals is attributable to intersite variations in proportions of at-risk subpopulations, number and effectiveness of outreach activities, geographic accessibility, and structural facilitators and/or barriers.

### Timeliness of Care

ACCESS-OM provided the majority of youths with an initial appointment within 72 hours and services within 30 days to an even larger proportion, both a priori benchmarks. This far exceeds the performance of traditional Canadian services.^17,22^ Delays to evaluations and services also shortened over time, particularly after year 1. Unlike in the literature,^41,42^ we found no social determinants associated with longer treatment delays. Direct, gatekeeper-free access to youth-friendly, nonphysician professionals trained in evaluating diverse presentations likely made intakes timelier. That timeliness can be improved even for disadvantaged youths should prompt policymakers and funders to insist on wait-time benchmarks.

Wait times are seldom reported for transformed youth services, partly because 0 delays are assumed for services that allow walk-ins, a common feature of transformed services including ACCESS-OM. However, even with walk-ins, there can be considerable waits.^43,44^ Our prospective, systematized measurement of wait times using recorded dates is likelier more accurate than the documentation of self-reported wait times in comparable intiatives.^45,46^

While mostly still within benchmark wait times, youths with moderate to severe mental health problems received initial offered appointments and services later. Delays were generally longer as severity of mental health problems increased. This is concerning because treatment delays are especially harmful for such youths.^47^ There may be longer wait times for specialist-provided services.^48^ Enhanced primary and/or community services (like ACCESS-OM) may not be designed or prepared to handle referrals for youths with severe mental health problems. Youth- and family-level factors (eg, stigma and social support) may also contribute to delays. While wait times were low throughout for the mildly ill, they improved for those with more severe problems. Although not significant, this interaction suggests that community-based services need a training or ramp-up phase to provide timely evaluation and services to those with more severe conditions.

While not significant, younger participants had longer wait-times. This may reflect developmental or familial differences and warrants investigation, as it has implications for age groupings that services should target or for ensuring equity across those aged 12 to 25 years.

## Implications

Our work has important policy implications as it bolsters evidence on the promising outcomes of low-barrier, primary and/or community-based, holistic youth mental health services that cater to youths with varying mental health presentations and needs. Youths with more severe mental health problems may not be benefiting equitably, indicating a need to investigate reasons for this, train such services to respond better and faster to this subgroup, and integrate better with specialist services.

### Strengths and Limitations

The lack of a control group and historical data limits conclusions about the effectiveness of our service transformation, a criticism leveled against several youth mental health reforms. Traditional RCTs may be inappropriate given the urgency of reform and the many external factors that affect feasibility. Nonetheless, our study is valuable because it tested a priori hypotheses. This helps establish with greater clarity how services in diverse settings change over time upon reformation.

While most youths received services before 30 days, we cannot tell how appropriate the services were for their problems. This concern was partly offset by staff being well-trained, but the services received may have been influenced by their availability, which varied across sites. We reported data on service categories and associated delays. Because services inappropriate for the illness stage (particularly when severe) can be ineffective or harmful,^49^ future research should evaluate the appropriateness of services along with their timeliness. About 30% received single-session therapy, problem solving, or care coordination. This approach is increasingly a feature of ACCESS-OM-like models, as it can be delivered during the first appointment. Future work should evaluate for whom this approach is suitable and sufficient. We evaluated illness severity based on CGI, not diagnoses. Because youths present with protean, overlapping mental health problems and sites were not based in research centers, stakeholders felt that intakes would be more practicable and acceptable if based on interviews complemented by measures (eg, transdiagnostic CGI) rather than diagnostic evaluations. Our approach helped characterize youths and identified some youths as having no mental health problems, which reduced unwarranted specialist care use.

We had data from only 67.8% of the referred youths who had an initial evaluation. We have no data on those referred but who could not be contacted or did not attend any appointment. The rest could not be contacted postreferral or did not attend an appointment. Our leakage level (32.1%) was lower than that of the 2 comparable studies to report it (Singapore: 46%^50^; Birmingham: 59%^36^).

Data were missing to different degrees across variables. We therefore used multiple imputation and pooled estimates to reduce loss of precision and selection biases introduced by complete-case analysis. To our knowledge, similar research has not typically reported on and appropriately handled missingness, a frequent concern in health services research.^51^

Being based in diverse primary and community settings enhanced our study’s external validity. Our future publications will investigate intrasite and intersite heterogeneity, as well as youth-level outcomes.

## Conclusion

Efforts to build better systems of care for youths must be guided by core principles and operationalized locally considering contextual and cultural factors.^3,12,52^ Our findings underscore the utility of approaches used in ACCESS-OM^26^ and learning health systems^53,54^ that engage organizations, communities, and lived-experience experts in identifying problems, designing services, implementing change, building capacities, and collecting and learning from common data. These insights are timely given the globally increasing investments in youth mental health care.

## Notes

Supplement. **eTable 1. **Handling Missing Data **eTable 2.** Description of Service Categories **eTable 3.** Distribution of First Services Received **eTable 4. **Results of the Accelerated Time Failure Models (comparing those with moderate to severe vis-à-vis mild and no to borderline mental health problems) **eTable 5. **Delay Between Referral and Initial Offered Appointment and Delay Between Initial Evaluation Appointment and First Service Received by Mental Health Problem Severity
eReferences.
**eFigure 1.** Benchmarks **eFigure 2.** Marginal Predicted Means and 95% CIs of Numbers of Referrals for Every 6-Month Progression in the Implementation of ACCESS Open Minds **eFigure 3.** Kaplan-Meier Curves for the First 3 Days from Referral to Offered Initial Appointment by Program Year **eFigure 4. **Kaplan-Meier Curves for the First 30 Days From Initial Evaluation to Service Provision by Program Year **eFigure 5.** Predicted Median Wait Times by Clinical Severity and Month since Program Implementation

Supplement 2.
Data Sharing Statement

## References

[1] Solmi M, Radua J, Olivola M, . Age at onset of mental disorders worldwide: large-scale meta-analysis of 192 epidemiological studies. Mol Psychiatry. 2022;27(1):281-295. doi:10.1038/s41380-021-01161-7 34079068 PMC8960395

[2] Erskine HE, Moffitt TE, Copeland WE, . A heavy burden on young minds: the global burden of mental and substance use disorders in children and youth. Psychol Med. 2015;45(7):1551-1563. doi:10.1017/S0033291714002888 25534496 PMC5922255

[3] McGorry PD, Mei C, Dalal N, . The Lancet Psychiatry Commission on youth mental health. Lancet Psychiatry. 2024;11(9):731-774. doi:10.1016/S2215-0366(24)00163-9 39147461

[4] Iyer SN, Boksa P, Lal S, . Transforming youth mental health: a Canadian perspective. Ir J Psychol Med. 2015;32(1):51-60. doi:10.1017/ipm.2014.89 31715701

[5] MacDonald K, Fainman-Adelman N, Anderson KK, Iyer SN. Pathways to mental health services for young people: a systematic review. Soc Psychiatry Psychiatr Epidemiol. 2018;53(10):1005-1038. doi:10.1007/s00127-018-1578-y 30136192 PMC6182505

[6] Malla A, Shah J, Iyer S, . Youth mental health should be a top priority for health care in Canada. Can J Psychiatry. 2018;63(4):216-222. doi:10.1177/0706743718758968 29528719 PMC5894919

[7] Iyer SN, Shah J, Boksa P, . A minimum evaluation protocol and stepped-wedge cluster randomized trial of ACCESS Open Minds, a large Canadian youth mental health services transformation project. BMC Psychiatry. 2019;19(1):273. doi:10.1186/s12888-019-2232-2 31488144 PMC6729084

[8] Fante-Coleman T, Jackson-Best F. Barriers and facilitators to accessing mental healthcare in Canada for Black youth: a scoping review. Adolesc Res Rev. 2020;5(2):115-136. doi:10.1007/s40894-020-00133-2

[9] Lines LA, Jardine CG; Yellowknives Dene First Nation Wellness Division. Connection to the land as a youth-identified social determinant of Indigenous Peoples’ health. BMC Public Health. 2019;19(1):176. doi:10.1186/s12889-018-6383-8 30744592 PMC6371607

[10] Goetz CJ, Mushquash CJ, Maranzan KA. An integrative review of barriers and facilitators associated with mental health help seeking among Indigenous populations. Psychiatr Serv. 2023;74(3):272-281. doi:10.1176/appi.ps.202100503 36065579

[11] Boksa P, Hutt-MacLeod D, Clair L, . Demographic and clinical presentations of youth using enhanced mental health services in six Indigenous communities from the ACCESS Open Minds Network. Can J Psychiatry. 2022;67(3):179-191. doi:10.1177/07067437211055416 34796730 PMC8935596

[12] Special Issue: ACCESS Open Minds: Transforming youth mental health services across Canada. Supplement 1. Wiley Online Library. Accessed January 27, 2025. https://onlinelibrary.wiley.com/toc/17517893/2019/13/S1

[13] Breuer E, Lee L, De Silva M, Lund C. Using theory of change to design and evaluate public health interventions: a systematic review. Implement Sci. 2016;11:63. doi:10.1186/s13012-016-0422-6 27153985 PMC4859947

[14] Guinaudie C, Mireault C, Tan J, . Shared decision making in a youth mental health service design and research project: insights from the Pan-Canadian ACCESS open minds network. Patient. 2020;13(6):653-666. doi:10.1007/s40271-020-00444-5 32996032 PMC7655783

[15] Levasseur MA, Roeszler L, den Besten L, Pinkoski K. Invited commentary: ACCESS Open Minds family and carers council. Early Interv Psychiatry. 2019;13(suppl 1):68-70. doi:10.1111/eip.12821 31243906

[16] Malla A, Iyer S, McGorry P, . From early intervention in psychosis to youth mental health reform: a review of the evolution and transformation of mental health services for young people. Soc Psychiatry Psychiatr Epidemiol. 2016;51(3):319-326. doi:10.1007/s00127-015-1165-4 26687237

[17] Kowalewski K, McLennan JD, McGrath PJ. A preliminary investigation of wait times for child and adolescent mental health services in Canada. J Can Acad Child Adolesc Psychiatry. 2011;20(2):112-119. 21541100 PMC3085670

[18] Jordan G, Kinkaid M, Iyer SN, . Baby or bathwater? Referrals of “non-cases” in a targeted early identification intervention for psychosis. Soc Psychiatry Psychiatr Epidemiol. 2018;53(7):757-761. doi:10.1007/s00127-018-1502-5 29541798

[19] Perestelo-Perez L, Gonzalez-Lorenzo M, Perez-Ramos J, Rivero-Santana A, Serrano-Aguilar P. Patient involvement and shared decision-making in mental health care. Curr Clin Pharmacol. 2011;6(2):83-90. doi:10.2174/157488411796151192 21592063

[20] MacDonald K, Malla A, Joober R, . Description, evaluation and scale-up potential of a model for rapid access to early intervention for psychosis. Early Interv Psychiatry. 2018;12(6):1222-1228. doi:10.1111/eip.12564 29582562

[21] Canadian Psychiatric Association. Wait time benchmarks for patients with serious psychiatric illnesses. 2006. Accessed February 3, 2025. https://www.cpa-apc.org/wp-content/uploads/Wait_times-CPA_policy_paper_1-web-EN.pdf

[22] Loebach R, Ayoubzadeh S. Wait times for psychiatric care in Ontario. University of Western Ontario Medical Journal. 2017;86(2):48-50. doi:10.5206/uwomj.v86i2.2027

[23] Hetrick SE, Bailey AP, Smith KE, . Integrated (one‐stop shop) youth health care: Best available evidence and future directions. Med J Aust. 2017;207(S10):S5-S18."https://pubmed.ncbi.nlm.nih.gov/28653978" doi:10.5694/mja17.00694 29129182

[24] The First Nations Information Governance Centre. Ownership, Control, Access and Possession (OCAP): the path to first nations information governance. 2014. Accessed January 16, 2025. https://fnigc.ca/wp-content/uploads/2020/09/5776c4ee9387f966e6771aa93a04f389_ocap_path_to_fn_information_governance_en_final.pdf

[25] Canadian Institutes of Health Research. TCPS 2—Chapter 9: research involving the First Nations, Inuit and Métis Peoples of Canada. 2019. Accessed January 16, 2025. https://ethics.gc.ca/eng/tcps2-eptc2_2018_chapter9-chapitre9.html

[26] Malla A, Iyer S, Shah J, ; ACCESS Open Minds Youth Mental Health Network. Canadian response to need for transformation of youth mental health services: ACCESS Open Minds (Esprits ouverts). Early Interv Psychiatry. 2019;13(3):697-706. doi:10.1111/eip.12772 30556335 PMC6563151

[27] Statistics Canada. 2016 Census of population long-form guide. 2016. Accessed February 3, 2025. https://www.statcan.gc.ca/en/statistical-programs/document/3901_D18_T1_V1

[28] Statistics Canada. Visible Minority and population group reference guide. Census of population, 2016. 2017. Accessed February 3, 2025. https://www12.statcan.gc.ca/census-recensement/2016/ref/guides/006/98-500-x2016006-eng.cfm

[29] Statistics Canada. Aboriginal peoples in Canada: key results from the 2016 census. 2017. Accessed February 3, 2025. https://www150.statcan.gc.ca/n1/daily-quotidien/171025/dq171025a-eng.htm?HPA=1

[30] Dunlop BW, Gray J, Rapaport MH. Transdiagnostic clinical global impression scoring for routine clinical settings. Behav Sci(Basel). 2017;7(3):40. doi:10.3390/bs7030040 28653978 PMC5618048

[31] RStudio Team. RStudio: Integrated Development for R. 2020. Accessed January 16, 2025. https://www.rstudio.com/.

[32] StataCorp. Stata Statistical Software: Release 18. 2023. Accessed January 16, 2025. https://www.stata.com/

[33] van Buuren SGOK. mice: Multivariate Imputation by Chained Equations in R. J Stat Softw. 2011;45(3):1-67. doi:10.18637/jss.v045.i03

[34] Statistics Canada. Census Profile. Census; 2016.

[35] Patulny R, Muir K, Powell A, Flaxman S, Oprea I. Are we reaching them yet? Service access patterns among attendees at the headspace youth mental health initiative. Child Adolesc Ment Health. 2013;18(2):95-102. doi:10.1111/j.1475-3588.2012.00662.x 32847285

[36] Birchwood M, Street C, Singh SP, . Impact and process evaluation of Forward Thinking Birmingham, the 0-25 Mental Health Service: final report. 2018. Accessed January 16, 2025. https://wrap.warwick.ac.uk/id/eprint/100545/

[37] Maxwell S, Ugochukwu O, Clarke T, . The effect of a youth mental health service model on access to secondary mental healthcare for young people aged 14-25 years. BJPsych Bull. 2019;43(1):27-31. doi:10.1192/bjb.2018.70 30236167 PMC6327291

[38] Statistics Canada. Table 98-10-0330-01 Visible minority by occupation, highest level of education and generation status: Canada, provinces and territories. 2023. Accessed January 16, 2025. https://www150.statcan.gc.ca/t1/tbl1/en/tv.action?pid=9810033001.

[39] Statistics Canada. Table 37-10-0196-01 Percentage of 15-to 29-year-olds in education and not in education by labour force status, highest level of education attained, age group and sex. 2023. Accessed January 16, 2025. https://www150.statcan.gc.ca/t1/tbl1/en/tv.action?pid=3710019601.

[40] Statistics Canada. Canada at a Glance. Accessed January 16, 2025. https://www150.statcan.gc.ca/n1/en/pub/12-581-x/12-581-x2022001-eng.pdf?st=nb8YNzDq.

[41] Steinman KJ, Shoben AB, Dembe AE, Kelleher KJ. How long do adolescents wait for psychiatry appointments? Community Ment Health J. 2015;51(7):782-789. doi:10.1007/s10597-015-9897-x 26108305

[42] Baciu A, Negussie Y, Geller A, Weinstein JN. Communities in action: pathways to health equity. 2017. Accessed January 16, 2025. https://nap.nationalacademies.org/catalog/24624/communities-in-action-pathways-to-health-equity

[43] O’Reilly A, O’Brien G, Moore J, . Evolution of jigsaw-a national youth mental health service. Early Interv Psychiatry. 2022;16(5):561-567. doi:10.1111/eip.13218 34464507

[44] headspace National Youth Mental Health Foundation. Increasing demand in youth mental health: a rising tide of need. April 5, 2019. Accessed January 16, 2025. https://headspace.org.au/our-organisation/media-releases/increasing-demand-in-youth-mental-health-a-rising-tide-of-need/

[45] Rickwood DJ, Telford NR, Mazzer KR, Parker AG, Tanti CJ, McGorry PD. The services provided to young people through the headspace centres across Australia. Med J Aust. 2015;202(10):533-536. doi:10.5694/mja14.01695 26021365

[46] Ishay GH, Zisman-Ilani Y, Roe D. A longitudinal study of headspace youth oriented mental health service satisfaction, service utilization and clinical characteristics. Early Interv Psychiatry. 2023;17(4):404-411. doi:10.1111/eip.13347 35981970

[47] Dell’osso B, Altamura AC. Duration of untreated psychosis and duration of untreated illness: new vistas. CNS Spectr. 2010;15(4):238-246. doi:10.1017/S1092852900000079 20414173

[48] KPMG. Evaluation of the national headspace program: final report to Department of Health. 2022. Accessed January 16, 2025. https://www.health.gov.au/resources/publications/evaluation-of-the-national-headspace-program?language=en

[49] Chanen AM, Betts JK, Jackson H, . Effect of 3 forms of early intervention for young people with borderline personality disorder: the MOBY randomized clinical trial. JAMA Psychiatry. 2022;79(2):109-119. doi:10.1001/jamapsychiatry.2021.3637 34910093 PMC8674805

[50] Harish SS, Kundadak GK, Lee YP, Tang C, Verma SK. A decade of influence in the Singapore youth mental health landscape: the Community Health Assessment Team (CHAT). Singapore Med J. 2021;62(5):225-229. doi:10.11622/smedj.2021061 34409464 PMC8801856

[51] Kisely S, Looi JC. Latest evidence casts further doubt on the effectiveness of headspace. Med J Aust. 2022;217(8):388-390. doi:10.5694/mja2.51700 36182662 PMC9826401

[52] Killackey E, Hodges C, Browne V, . A global framework for youth mental health: investing in future mental capital for individuals, communities and economies. 2020. Accessed January 16, 2025. https://www3.weforum.org/docs/WEF_Youth_Mental_Health_2020.pdf

[53] Heinssen RK, Azrin ST. A national learning health experiment in early psychosis research and care. Psychiatr Serv. 2022;73(9):962-964. doi:10.1176/appi.ps.20220153 35895842 PMC9444914

[54] Institute of Medicine Roundtable on Value and Science Driven Care. Integrating research and practice: health system leaders working toward high-value care: workshop summary. The National Academies Press. 2015. Accessed February 3, 2025. https://nap.nationalacademies.org/catalog/18945/integrating-research-and-practice-health-system-leaders-working-toward-high 25834870

