# Supplementary material for: An Approach to Providing Timely Mental Health Services to Diverse Youth Populations
Source: JAMA Psychiatry. 2025 Feb 26;82(5):470–80. doi: 10.1001/jamapsychiatry.2024.4880 (PMC11866065; doi:10.1001/jamapsychiatry.2024.4880)
Supplement: Supplement. — eTable 1. Handling Missing Data eTable 2. Description of Service Categories eTable 3. Distribution of First Services Received eTable 4. Results of the Accelerated Time Failure Models (comparing those with moderate to severe vis-à-vis mild and no to borderline mental health problems) eTable 5. Delay Between Referral and Initial Offered Appointment and Delay Between Initial Evaluation Appointment and First Service Received by Mental Health Problem Severity eReferences. eFigure 1. Benchmarks eFigure 2. Marginal Predicted Means and 95% CIs of Numbers of Referrals for Every 6-Month Progression in the Implementation of ACCESS Open Minds eFigure 3. Kaplan-Meier Curves for the First 3 Days from Referral to Offered Initial Appointment by Program Year eFigure 4. Kaplan-Meier Curves for the First 30 Days From Initial Evaluation to Service Provision by Program Year eFigure 5. Predicted Median Wait Times by Clinical Severity and Month since Program Implementation [file jamapsychiatry-e244880-s001.pdf]

Supplemental Online Content

Iyer SN, Boksa P, Joober R, et al. An approach to providing timely mental health services to diverse youth populations. *JAMA Psychiatry*. doi:10.1001/jamapsychiatry.2024.4880

eTable 1. Handling Missing Data

eTable 2. Description of Service Categories

eTable 3. Distribution of First Services Received

eTable 4. Results of the Accelerated Time Failure Models (comparing those with moderate to severe vis-à-vis mild and no to borderline mental health problems)

eTable 5. Delay Between Referral and Initial Offered Appointment and Delay Between Initial Evaluation Appointment and First Service Received by Mental Health Problem Severity

eReferences.

eFigure 1. Benchmarks

eFigure 2. Marginal Predicted Means and 95% CIs of Numbers of Referrals for Every 6-Month Progression in the Implementation of ACCESS Open Minds

eFigure 3. Kaplan-Meier Curves for the First 3 Days from Referral to Offered Initial Appointment by Program Year

eFigure 4. Kaplan-Meier Curves for the First 30 Days From Initial Evaluation to Service Provision by Program Year

eFigure 5. Predicted Median Wait Times by Clinical Severity and Month since Program Implementation

This supplemental material has been provided by the authors to give readers additional information about their work.

© 2025 Iyer SN et al. *JAMA Psychiatry*.

***eTable 1*. Handling Missing Data**

Reason for missingness	As is presented in Table 1, the prevalence of missing data ranged from 4% -50% depending on the variable. The amount of missingness is unsurprising as data for this study was collected using a mixture of administrative records, self-report measures and clinical interviews. The demographic form for example, contained many detailed questions which may not have been available via administrative records. While young people and stakeholders participated in the design of the questionnaires, which would mitigate selective non-response, it is possible that some young people did not feel comfortable answering some of the questions, resulting in missingness. Finally, while data collected via clinical interviews were overall more complete, some clinicians may have been selective in questions they asked young people depending on the case. Some sites also expressed concerns over the amount of data to collect and began to prioritize certain scales over others. This resulted in more complete data on some items compared to others.
Consequences of missingness	While examining the data, patterns of missingness were identified. These patterns suggested that the missing completely at random (MCAR) assumption was violated and performing a complete case analysis could result in biased estimates. Since information on a variety of variables including demographics, clinical and service use characteristics was collected, it was reasonable to assume the data was missing at random (MAR) and could therefore be handled with imputation.
Method for handling missingness	Since multiple variables, and different types of variables in the dataset had missingness, the multiple imputation was performed using multivariate imputation by chained equations (MICE). Assuming that data is missing at random (MAR), this method accounts for variability in the imputing process and retains relationships that exist pre-imputation[1]. Separate imputations were run for hypothesis two and three since it was a slightly different sample (i.e., for hypothesis three, only participants who were ‘at risk’ of receiving a service were included). Unlike the imputation for hypothesis two, time from initial assessment to service provision was imputed for hypothesis three. All time variables were imputed as cumulative baseline hazards and back translated to times post imputation.
Software and relevant packages	The mice[1] package in R was used to run the MICE. The method of imputation varied by the variable type; binary variables were imputed via logistic regression imputation (logreg), continuous variables were imputed by predictive mean matching (pmm), categorical variables were imputed via polytomous regression (polyreg) and ordinal variables were imputed with proportional odds models (polr). Blocks were visited from left to right and the predictor matrix was set using the quickpred function with correlation 0.1. The predictor matrix was modified so that site was a predictor in all imputations to account for site level differences. The only variables which were not allowed to predict one another were time from referral to first in person appointment and time from referral to first offered appointment due to multicollinearity. Sixty datasets were imputed since there was up to 60% missingness of some of the variables involved in the imputation (some were auxiliary variables). The number of iterations was also set to 60 and

© 2025 Iyer SN et al. *JAMA Psychiatry*.

	was determined using trial and error while assessing convergence of each variable across the imputed datasets.
Who is missing outcome data for Hypothesis 2?	The outcome of interest for hypothesis two, time from referral to appointment offer was missing for 396 participants. Among these participants, 8 were missing age, 16 were missing gender, 194 were missing ethnic group, 186 were missing NEET status, 321 were missing ability to meet basic needs, 341 were missing sexual orientation and 223 were missing CGI score. Across the remaining 3960 youth with data on this outcome, the proportion of youth missing age, gender, ethnic group, NEET, and CGI score was higher for those offered an appointment within 72 hours of being referred; 85% of those missing age (n= 158/186), 88% of those missing gender (n= 218/247), 56% of those missing ethnic group (n= 658/1167), 79% of those missing NEET status (n=1238/1572), and 58% of those with a missing CGI score (507/874). While the proportion of youth missing ability to meet basic needs and sexual orientation was higher for those who were not offered an appointment within 72 hours of being referred; 50% of those missing ability to meet basic needs (n=926/1848), 54% of those missing sexual orientation (n=902/1671).
Who is missing outcome data for Hypothesis 3?	The outcome of interest for hypothesis three, time from initial evaluation to service received, was missing for 666 participants. Among these participants, 5 were missing age, 6 were missing gender, 205 were missing ethnic group, 207 were missing NEET status, 435 were missing ability to meet basic needs, 449 were missing sexual orientation and 269 were missing CGI score. Across the remaining 3085 youth with data on this outcome, the proportion of youth missing one of the covariates mentioned above was consistently higher among those who received services within 30 days of their initial assessment; 100% of those missing age (n=168/168), 99% of those missing gender (n=212/213), 94% of those missing ethnic group (n=881/940), 98% of those missing NEET status (n=1219/1238), 91% of those missing ability to meet basic needs (n=1298/1421), 92% of those missing sexual orientation (n=1185/1287), and 98% of those with a missing CGI score (479/490).

© 2025 Iyer SN et al. *JAMA Psychiatry*.

***eTable 2.* Description of Service Categories**

Service Category	Description/examples
Individual therapy	General/supportive counselling, specific individual counseling/therapy/interventions, problem solving
Group therapy	Trauma group, anxiety group, general group therapy
Psychoeducation	Providing information for youth to help them understand and cope with their mental health problem.
Self-help resources	Handouts, books, self-care
Mental health: Psychosocial	Skills training, social worker support unspecified, occupational therapy, support unspecified, drop-in unspecified, wellness activities
Mental health: Psychiatric	Psychiatric consultation, consultation with family physician/nurse practitioner specializing in mental health
Mental health: Specialized Service package	Service package for complex or serious mental illness (e.g., specialized early intervention for psychosis) or specific populations
Crisis intervention	Crisis team, risk management, safety plan
Case management/care coordination	Case management, care coordination
Online/e Mental health/helpline	Mental health supports delivered online, by phone, or through smartphone apps
Hospitalization/Emergency room	Emergency room visit, inpatient
Alcohol and/or drug-specific intervention	Detoxification, interventions to reduce substance use
Physical health	Physical health interventions/supports, consultation with physician/nurse/nutritionist
Family/carer interventions	Family/carer psychoeducation/counselling
Peer support	Youth and family peer support, self-help groups
Focus on work/school and primary needs	Primary needs (housing, financial assistance), employment, education supports
Gender/sexuality and sexual health supports	Sexual health counselling, health services for gender transition
Evaluation Assessment	Psychiatric evaluation, needs assessment

© 2025 Iyer SN et al. *JAMA Psychiatry*.

***eTable 3.* Distribution of First Services Received**

Service	N (youth)*
Individual therapy	990
Solution focused single session and care coordination/ support/problem-solving provided during the initial assessment	908
Case management/care coordination	680
Mental health: Psychosocial	253
Peer support	189
Physical health	132
Mental health: Psychiatric	90
Mental health: Specialized Service package	75
Focus on work/school and primary needs	74
Group therapy	71
Psychoeducation	62
Evaluation Assessment	39
Crisis intervention	33
Alcohol and/or drug-specific intervention	34
Gender/sexuality and sexual health supports	13
Self-help resources	12
Family/carer interventions	11
Referral/navigation	10
Hospitalization/Emergency room	8
Other services	7
Online/eMental health/helpline	4

Data on this variable was missing for 666 youth. Of the remaining 3085 young people, 2568 received only one service while 517 received more than one service on the earliest service date; 433 received 2 services, 76 received 3 services, 7 received 4 services and 1 received 5 services. This table therefore represents 3695 services received by the 3085 young people with data on this variable.

© 2025 Iyer SN et al. *JAMA Psychiatry*.

***eTable 4.* Results of the Accelerated Time Failure Models (comparing those with moderate to severe vis-à-vis mild and no to borderline mental health problems)**

	Time from referral to first offered evaluation appointment	Time from first appointment to first service received
Variable	Adjusted TR (95%CI)	Adjusted TR (95%CI)
Referral period		
Per 6-months increment	**0.98 (0.96-1.00)**	**0.97 (0.94-1.00)**
Age (years, continuous)	0.99 (0.98-1.01)	1.01 (0.99-1.03)
Gender		
Cis woman	Ref.	Ref.
Cis man	0.99 (0.91-1.08)	1.00 (0.91-1.10)
Gender-diverse	1.02 (0.86-1.20)	1.08 (0.91-1.29)
Ethnic or cultural origins		
White	Ref.	Ref.
Visible minority	0.97 (0.86-1.09)	0.97 (0.86-1.10)
Indigenous	1.03 (0.88-1.19)	0.92 (0.79-1.07)
Education, employment, or training status		
Engaged in education, employment, or training	Ref.	Ref.
Not in education, employment, or training (NEET)	0.93 (0.82-1.05)	0.99 (0.89-1.10)
Ability to meet basic needs		
No difficulty meeting basic needs	Ref.	Ref.
Difficulty meeting basic needs	0.97 (0.85-1.11)	0.97 (0.86-1.09)
Sexual orientation		
Heterosexual	Ref.	Ref.
Sexual minority	1.05 (0.94-1.18)	1.06 (0.95-1.17)
Severity of mental health problem(s)		
Moderate to severe	Ref.	Ref.
None to borderline	**0.85 (0.74-0.97)**	**0.86 (0.74-1.00)**
Mild	**0.88 (0.79-0.97)**	0.92 (0.83-1.03)

© 2025 Iyer SN et al. *JAMA Psychiatry*.

TR, time-ratio; 95%CI, 95% confidence interval; NEET, not in education, employment or training; CGI, Clinical Global Impression scale. Coefficients in bold represent statistically significant associations. All models were mixed effects multilevel models with random intercepts by site (N=11).

© 2025 Iyer SN et al. *JAMA Psychiatry*.

eTable 5. **Delay Between Referral and Initial Offered Appointment and Delay Between Initial Evaluation Appointment and First Service Received by Mental Health Problem Severity***

	**Days to Appointment Offer**			**Days to Service Received**		
	Total N =4356			Total N=3751		
**Mental health problem severity**	N (%)	Mean days (SD)	Median (IQR)	N (%)	Mean days (SD)	Median (IQR)
No to borderline mental health problem	584 (13.40%)	9.56 (23.10)	1.43 (9.15)	507(13.52%)	12.56 (49.17)	0.00 (0.00)
Mild mental health problem	986 (22.64%)	8.85 (24.64)	0.00 (7.33)	837 (22.31%)	12.54 (43.19)	0.00 (0.00)
Moderate to severe mental health problem	2786 (63.95%)	11.28 (30.83)	0.00 (7.97)	2407 (64.17%)	16.64 (53.39)	0.00 (0.20)

**All values are pooled over 60 imputed datasets which is why not all percentages will add to 100.*

© 2025 Iyer SN et al. *JAMA Psychiatry*.

eReferences

1. van Buuren, S., & Groothuis-Oudshoorn, K. (2011). mice: Multivariate Imputation by Chained Equations in R. Journal of Statistical Software, 45(3), 1–67. https://doi.org/10.18637/jss.v045.i03

© 2025 Iyer SN et al. *JAMA Psychiatry*.

eFigure 1. Benchmarks

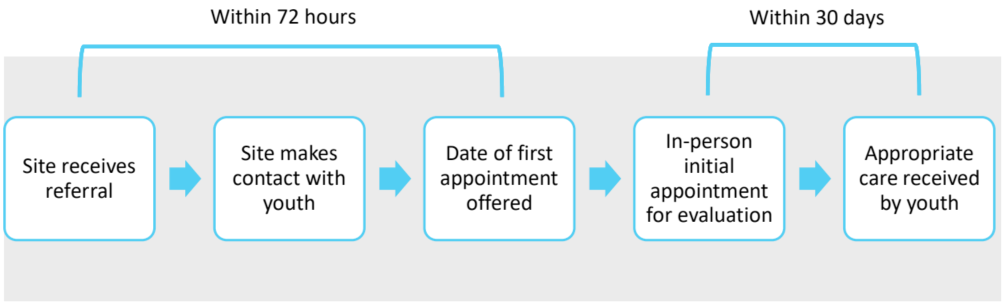

Supplemental Figure: Benchmarks set by ACCESS Open Minds. Referrals came from multiple sources, including young people themselves, families, peers, doctors, nurses, community organizations, school counselors, social workers, Elders, day shelters, and campus resources.

© 2025 Iyer SN et al. *JAMA Psychiatry*.

eFigure 2. Marginal Predicted Means and 95% CIs of Numbers of Referrals for Every 6-Month Progression in the Implementation of ACCESS Open Minds

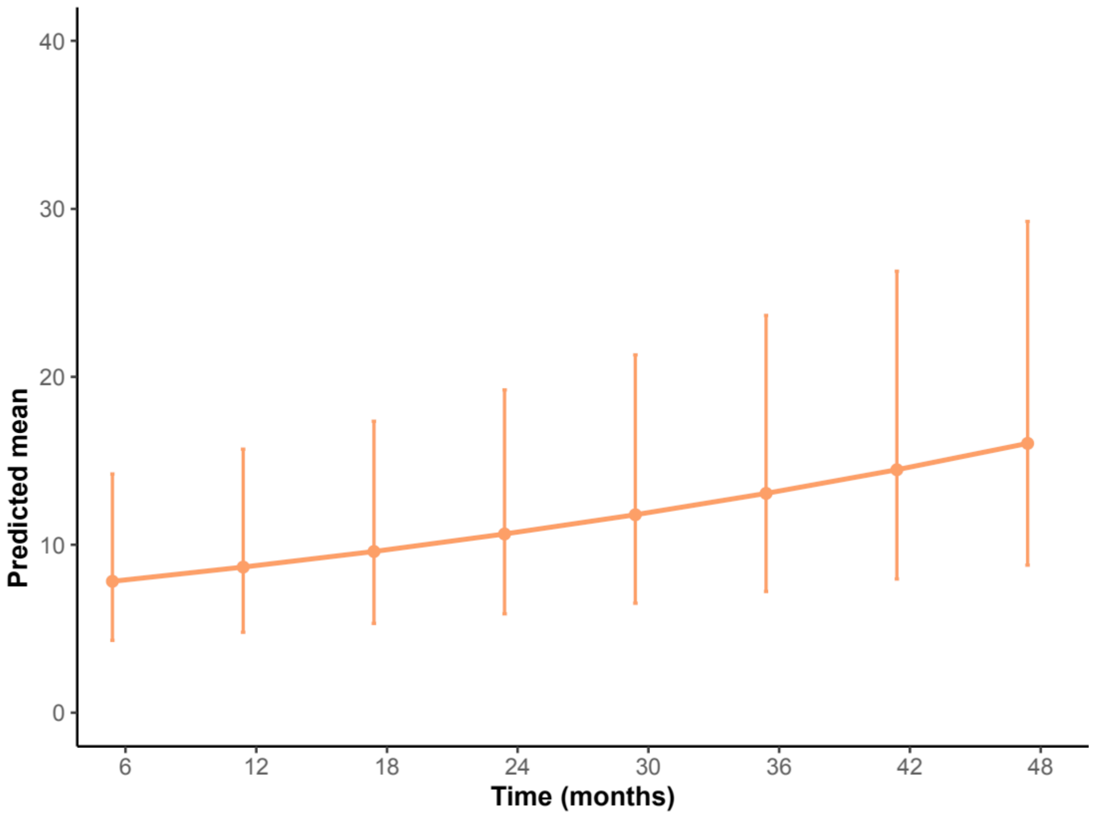

Note: Time since implementation at each site varied between 29 and 48 months

© 2025 Iyer SN et al. *JAMA Psychiatry*.

eFigure 3. Kaplan-Meier Curves for the First 3 Days From Referral to Offered Initial Appointment by Program Year

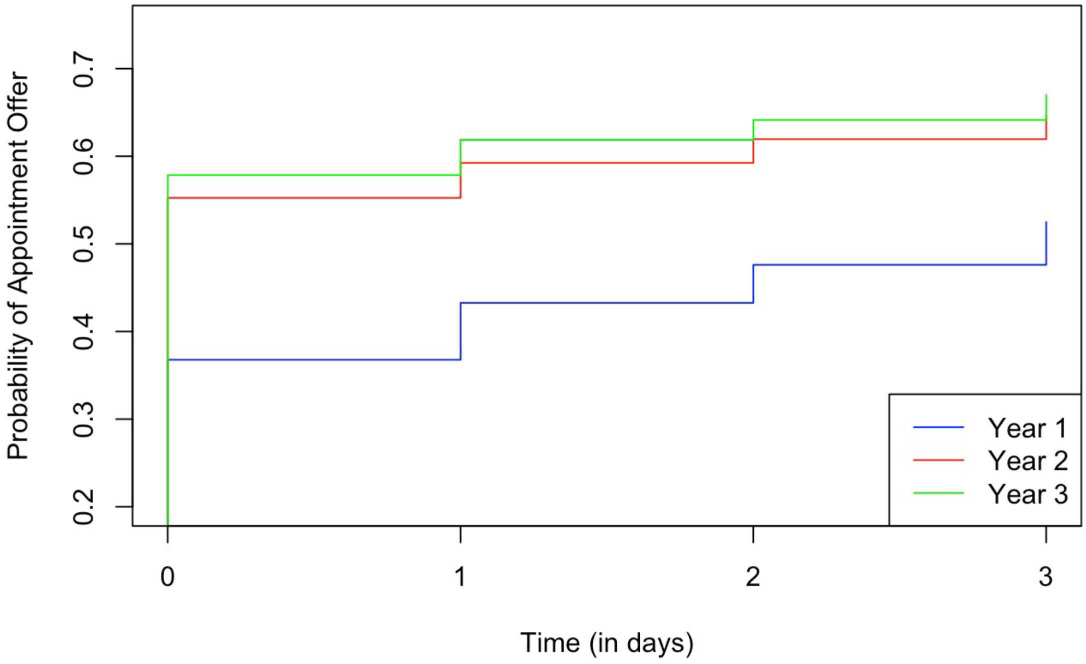

Note: Number at risk was 861, 1335, and 1545 in program years 1, 2, and 3, respectively. No participants were censored.

© 2025 Iyer SN et al. *JAMA Psychiatry*.

eFigure 4. Kaplan-Meier Curves for the First 30 Days From Initial Evaluation to Service Provision by Program Year

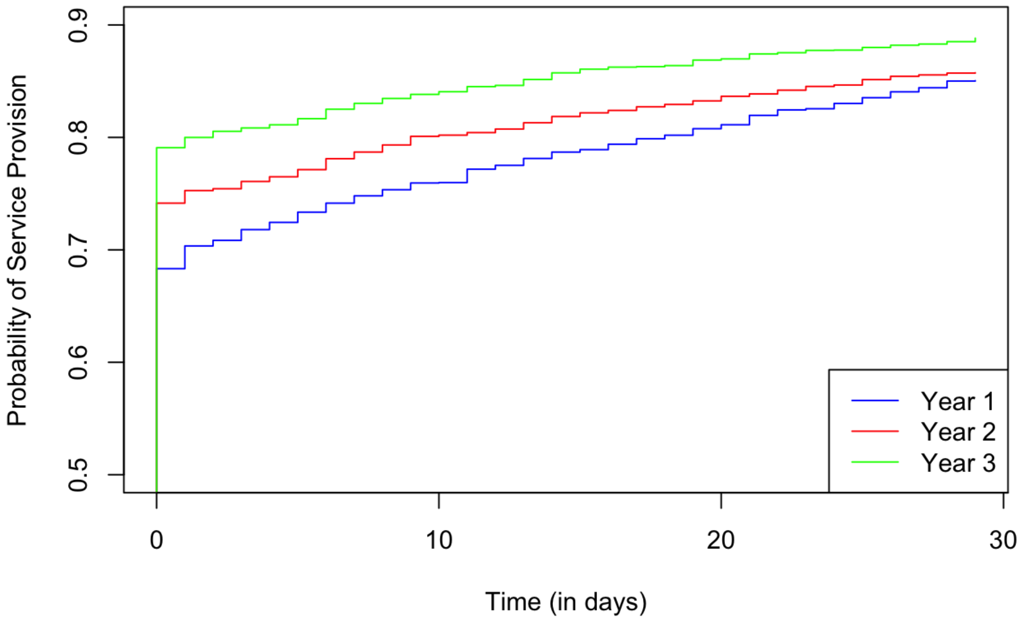

Note: Number at risk was 729, 1122, and 1406 in program year 1, 2, and 3, respectively. No participants were censored.

© 2025 Iyer SN et al. *JAMA Psychiatry*.

eFigure 5. Predicted Median Wait Times by Clinical Severity and Month Since Program Implementation

eFigure 5A. Predicted median wait time (in days) from referral/help-seeking to first offered appointment by clinical severity and month since program implementation

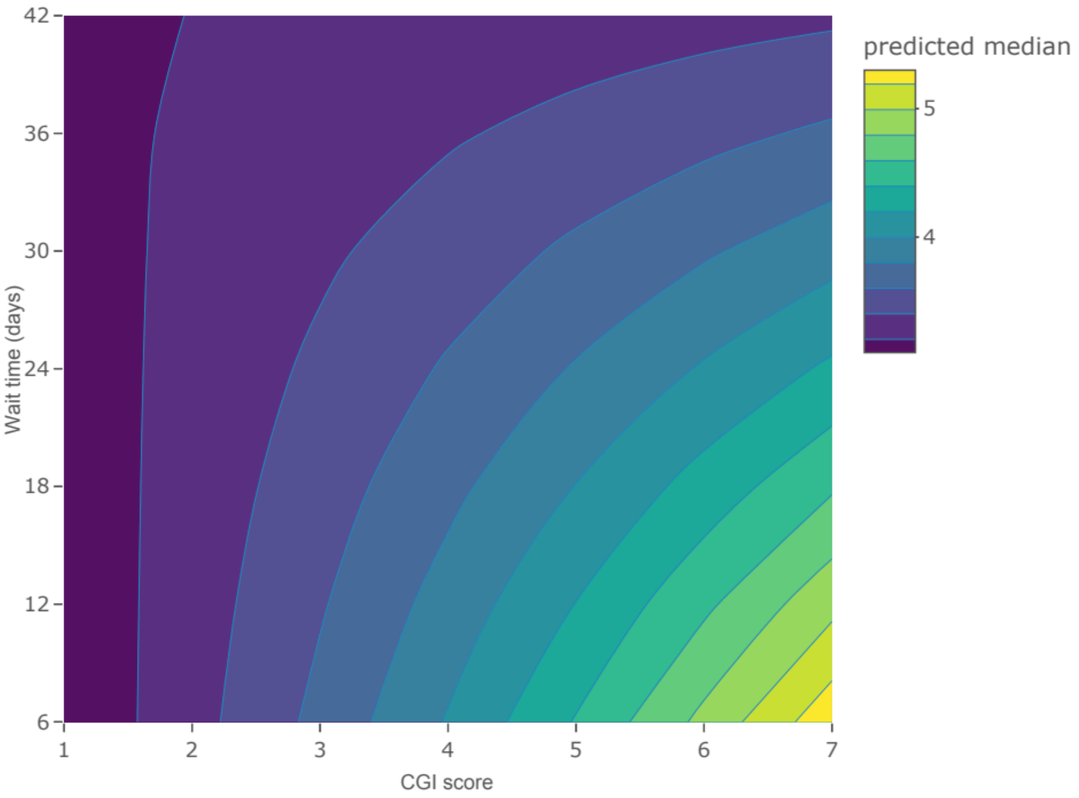

© 2025 Iyer SN et al. *JAMA Psychiatry*.

eFigure 5B. Predicted median wait time (in days) from initial evaluation to first received service by clinical severity and month since program implementation

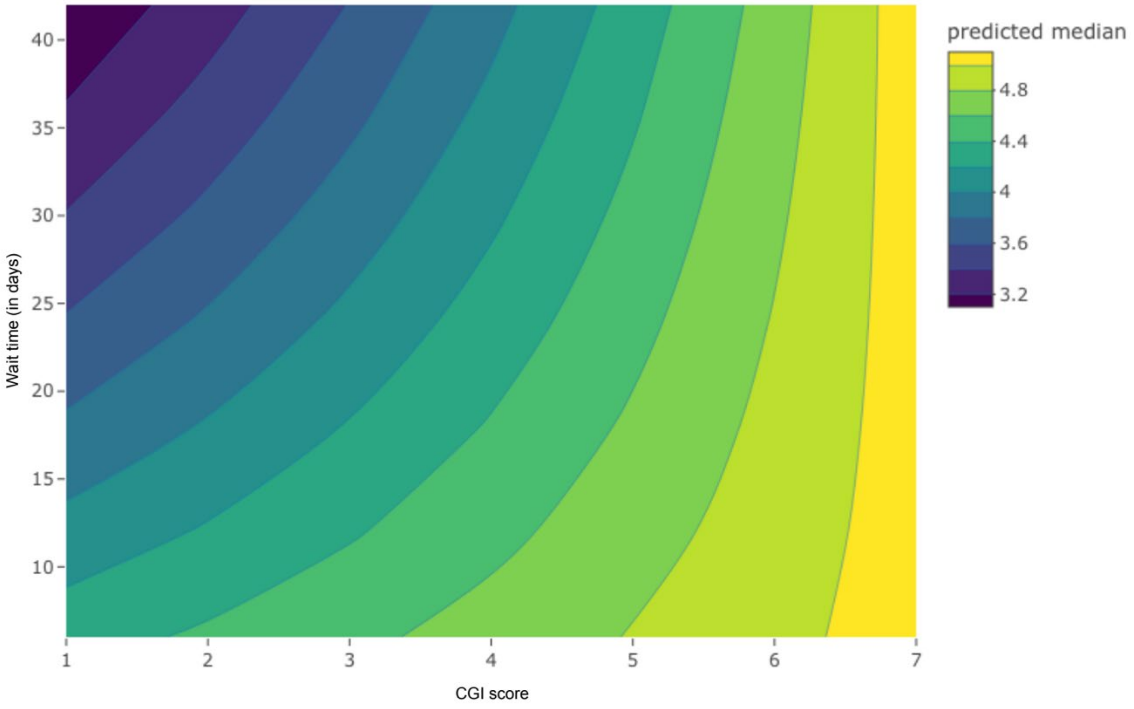

© 2025 Iyer SN et al. *JAMA Psychiatry*.
