# Supplementary material for: An Approach to Providing Timely Mental Health Services to Diverse Youth Populations
Source: JAMA Psychiatry. 2025 Feb 26;82(5):470–80. doi: 10.1001/jamapsychiatry.2024.4880 (PMC11866065; doi:10.1001/jamapsychiatry.2024.4880)
Supplement: Supplement 2. — Data Sharing Statement [file jamapsychiatry-e244880-s002.pdf]

Data Sharing Statement

Iyer. Providing Timely Access to Diverse Youth Mental Health Services. *JAMA Psychiatry*. Published February 19, 2025. doi:10.1001/jamapsychiatry.2024.4880

Data
Data available: Yes
Data types: Data dictionary
How to access data: srividya.iyer@mcgill.ca
When available: With publication

Supporting Documents
Document types: None

Additional Information
Who can access the data: researchers whose proposed use of the data has been approved
Types of analyses: To be discussed
Mechanisms of data availability: with co-investigator support, after approval of a proposal, and with a signed data access agreement
